# Astaxanthin is neuroprotective in an aged mouse model of Parkinson’s disease

**DOI:** 10.18632/oncotarget.23737

**Published:** 2017-12-28

**Authors:** Beth Grimmig, Lauren Daly, Meena Subbarayan, Ched Hudson, Robert Williamson, Kevin Nash, Paula C. Bickford

**Affiliations:** ^1^ Center of Excellence for Aging and Brain Repair, Department of Neurosurgery and Brain Repair, USF Morsani College of Medicine, Tampa, 33612 FL; ^2^ Department of Molecular Pharmacology and Physiology, USF Morsani College of Medicine, Tampa, 33612 FL; ^3^ Research Service, James A Haley Veterans Hospital, Tampa, 33620 FL; ^4^ Sanford Burnham Prebys Medical Discovery Institute at Lake Nona, Orlando, 32827 FL; ^5^ USF Health Byrd Alzheimer's Institute, Morsani College of Medicine, University of South Florida, Tampa, 33613 FL

**Keywords:** astaxanthin, neuroprotection, neurodegeneration, neuroinflammation, aging

## Abstract

Parkinson's disease (PD) is the second most common neurodegenerative disorder and prevalence increases with age. Normal physiological changes that occur during the aging process reflect the pathological characteristics of Parkinson's disease. It is also recognized that age related changes significantly interact with the pathological mechanisms that underlie the neurodegeneration in PD and perpetuate the disease process. Despite the fact that aging is considered to be a primary risk factor for developing PD, the use of aged animal models are still under-utilized in pre-clinical research, thus reducing the translatability of experimental findings. Here, we use a natural compound astaxanthin (AXT) with multiple biological activities to attenuate neurotoxicity in a mouse model of Parkinson's disease in both young and aged mice. We observed that AXT preserved neurons in the substantia nigra of both young and aged mice that were exposed to the MPTP neurotoxin. However, AXT was less efficacious in the aged animals, as AXT was not able to protect against the MPTP induced loss of tyrosine hydroxylase (TH) throughout the aged nigro-striatal circuit. This disparity in the neuroprotective effect of AXT suggests that aging is a critical factor to consider during the development of novel therapeutics for neurodegenerative diseases and should be more rigorously evaluated in preclinical models.

## INTRODUCTION

Parkinson's disease (PD) is a common neuro degenerative disorder that is primarily characterized by motor impairments including postural instability and bradykinesia. These motor symptoms are caused by loss of dopaminergic neurons in the substantia nigra pars compacta (SNpc) resulting in degeneration of those projections to the striatum. However, there is also notable dysfunction in other neurotransmitter systems and brain regions that account for a cluster of non-motor symptoms as well. Like other neurodegenerative conditions, PD is associated with aging. While there are some cases that arise from a crippling genetic predisposition, only 5% of diagnosis are genetically linked; the rest are idiopathic and have no known cause. Aging is considered to be the most substantial risk factor for developing PD, evidenced by the increase in prevalence for every decade after 60. Moreover, it is projected that by 2050, the US population over the age of 65 will double [1]. The prevalence of this disorder is predicted to increase dramatically, as well as the associated economic burden of long term health care. Current medications primarily alleviate motor symptoms, while the debilitating non-motor symptoms continue to reduce quality of life for these patients. There are no treatments available that address the neurodegeneration or restore the loss of neurons in the SNpc. Therefore, development of more effective drugs still represents a major and unfulfilled goal of this field. Interestingly, there have been many compounds identified in preclinical settings that demonstrated neuroprotective properties and the prospect of being developed into useful therapeutic agents. However, many of these compounds have failed in clinical trials. There has been some speculation around this translational disappointment, and recent discussion has begun to critically evaluate the animal models used to test novel therapeutic agents and their utility in translation. While each experimental model has its own pros and cons, their value and utility are dependent on the experimental design and endpoints, as reviewed here [2]. However, one glaring criticism of experimental models is the lack of use of aged animals in many of these conditions. Aging is an essential element involved in Parkinson's disease; we can clearly see this relationship in the exponential increase in prevalence of PD in the advanced decades [3]. Even normal healthy aging in the absence of pathology involves many physiological changes. These age-related changes are especially relevant to the onset of PD, as they are pronounced in the substantia nigra pars compacta, the region that degenerates in PD and is responsible for the motor impairment. For example, it is known that these DA cells in the nigra die off with advancing age at estimated rate of 10% per decade [4]. Furthermore, age related DA cell loss shows a similar substructural vulnerability within the SNpc that reflects the patterns of degeneration seen in patients diagnosed with PD. In both normal aging, and in confirmed cases of PD, there is selective cell loss in the ventral tier of the SNpc. Moreover, these aged DA cells also show additional markers of PD pathology, including accumulation of synuclein within the cytoplasm, increased levels of 3 nitrotyrosine (indicating oxidative and nitrative stress) and activation of both astrocytes and microglia in the vicinity [5]. It has also been observed that some elderly individuals have Lewy body pathology, the extent of which positively correlated with the severity of motor impairment. None of these patients were diagnosed with PD, but together these observations suggest that normal aging creates a susceptibility and similarity to PD pathology [6, 7]Interestingly, the substantia nigra has unique physiological characteristics that distinguish this region from similar dopaminergic centers and render this structure vulnerable to generic brain wide insults [8, 9]. This is largely due to high inflammation and oxidative stress. For example, the SN has a high population of microglia [10], which likely renders this region highly susceptible to inflammatory events which would have a heightened impact on the neurons of this region. Additionally, the SN produces unusually high levels of ROS and has low levels of glutathione, both of which contribute to oxidative damage and therefore cellular stress in this region [11, 12].

The pathophysiology of PD is critically important to consider within the context of aging. The normal aging process inevitably leads to physiological changes, some of which interact with and exacerbate the pathological mechanisms that underlie the neurodegeneration. For example, proteostaisis and autophagy, mitochondrial function, production and resolution of reactive oxygen species are all biological process that lose efficacy during normal aging that are also proposed to contribute to cell death in PD [13]. During normal aging in humans there is a steady loss of cells in the SNpc at rate of approximately 7% per decade [14]. Interestingly, there are many biochemical similarities that lead to loss of function in this region between normal aging and Parkinson's disease. For example, in both cases there is a selective vulnerability of the ventral tier of the SNpc, where dopaminergic cells are most impacted. Analysis of the aged non-human primate brain reveals that there is a marked loss of tyrosine hydroxylase (TH) within the ventral midbrain. The pattern of TH loss also reflects the vulnerability of subregions of dopaminergic neurons, where the age-related decrease in TH intensity is most pronounced in the ventral tier of the SNpc. There is simultaneously a marked accumulation of α-synuclein within the soma of the remaining cells that correlates with TH loss. It has been postulated that aging leads to a pre-parkinsonian state [5, 14]. Neuroinflammation also increases with age and is an integral aspect of the disease process. One relevant age related change that contributes to increased neuroinflammation is the priming of microglia, where these cells become hyperresponsive to stimulation and remain activated for longer durations as they simultaneously become less resistant to endogenous regulatory signaling [15, 16]. This altered microglial response increases and prolongs the parenchyma to cytotoxic cytokines and reactive oxygen species (ROS) [17].

Carotenoids have historically been investigated for their impact on human health. Certain carotenoids serve as pre-cursors for the synthesis of vitamin A, an essential biological activity in humans, while other non-provitamin A carotenoids have additional salubrious effects like protection from photo damage [18, 19]. Astaxanthin is a carotenoid that is produced by photosynthetic microorganisms. It is approved by the Food and Drug Administration as an antioxidant, and marketed as a health supplement. This compound has recently gained a lot of interest for its variety of health promoting effects [20–22]. It is being investigated as a treatment for a variety of clinical conditions, generating data from diverse model organisms that collectively suggest that this compound has multiple mechanisms of action [21]. Interestingly, these proposed mechanisms of action seem to counteract the pathological mechanisms that contribute to cell loss in PD. For example, AXT has been suggested to modulate immune function including microglial activation [23–27], enhance mitochondrial function [19, 28, 29] and is known to be an effective antioxidant [30–33]. We have previously shown that AXT supplementation is neuroprotective against MPTP in young animals, preserving the expression of tyrosine hydroxylase in the nigrostriatal tract, reducing microglial activation and enhancing the antioxidant capacity in AXT treated mice [34]. Pharmacokinetics confirm that AXT can cross the blood brain barrier, as its chemical properties of hydrophobicity would suggest. AXT will associate with lipid dense cellular membranes and predominantly accumulate in the liver, although it is known to also accumulate in the brain [35]. Recent reports support that AXT can exert its activity within the CNS and demonstrate neuroprotective mechanisms against multiple neuronal insults. For example, AXT has been suggested to enhance neuroplasticity and cognitive function [36]. Additionally, AXT supplementation was shown to be protective against traumatic brain injury, preserving cortical neurons and reducing subsequent behavioral deficits [37]. Likewise, AXT treatment also protected against neuron loss in a model of subarachnoid hemorrhage [30]. Recent experimentation further supports the efficacy of AXT *in vivo*, lending support to the concept of AXT based therapeutic strategies in neurological conditions [30, 38, 39].

We have previously shown AXT supplementation can reduce the neurotoxicity of MPTP in mice 3 months of age [34]. However, it is important to note that many compounds that have been successful pre-clinically have largely failed in clinical trials and a need for novel agents that can halt the neurodegenerative process remains unmet. Given that aging is in fact the biggest predictor of developing PD, the field has started to focus on the value of aged experimental animal models, with the thought that the physiology in aged animals is a more relevant backdrop to the human condition, thus possibly lending to more predictive validity [40]. Therefore, here we investigate the efficacy of AXT supplementation against MPTP neurotoxicity in aged animals.

## RESULTS

### AXT rescues MPTP induced neurodegeneration

**Figure 1:**
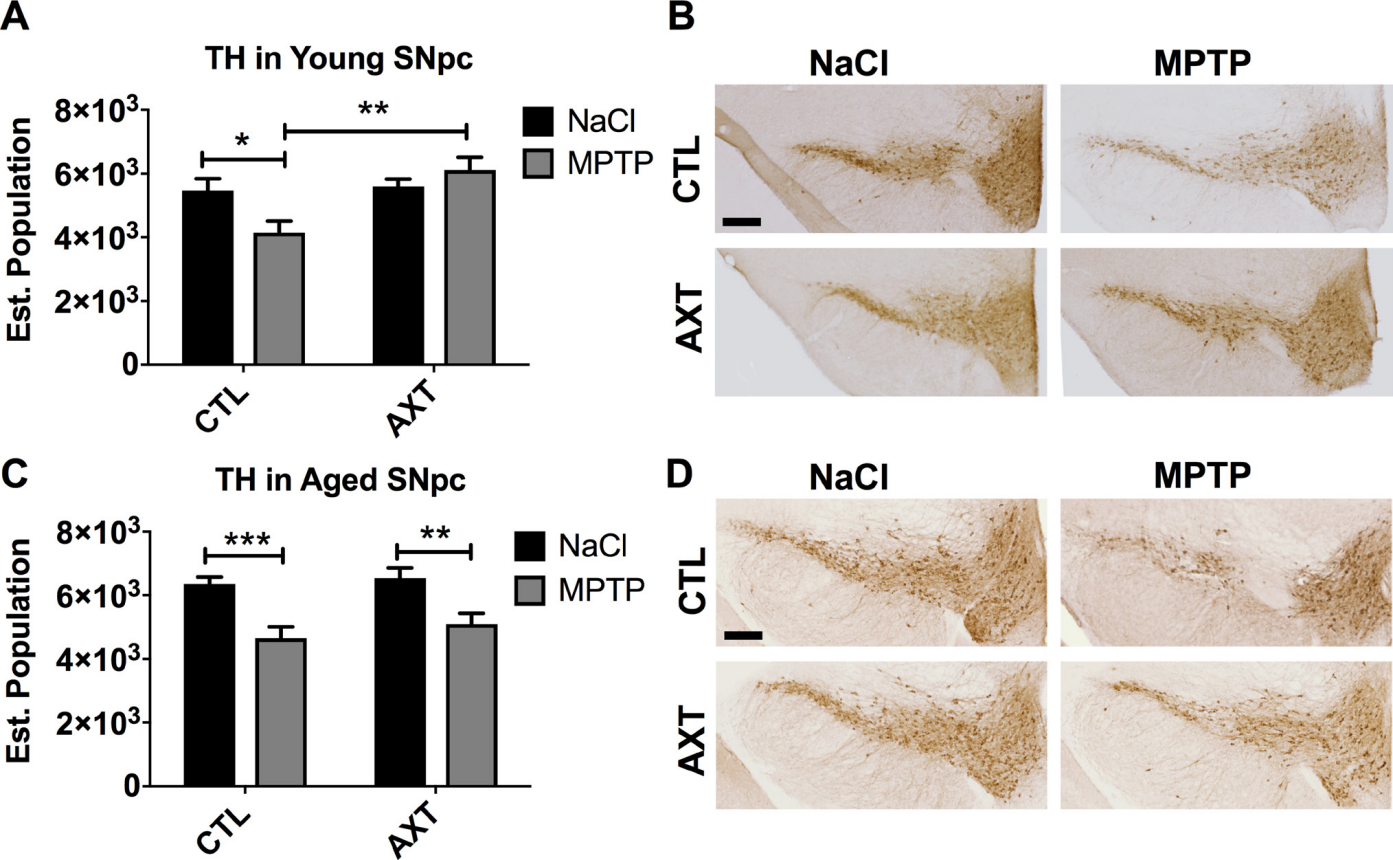
Consumption of the AXT enriched diet attenuates the loss of TH positive neurons in the substantia nigra pars compacta in young mice (**A**, **B**) while aged animals do not demonstrate any protection against MPTP induced neurodegeneration (**C**, **D**). (A) Bar graph of stereological quantification of TH positive neurons in the SNpc in young and (C) aged mice. Data is represented as mean + SEM. Immunohistochemistry with anti-TH antibody in the SNpc of (B) young and (D) aged mice. 2 way ANOVA; Young: *p* < 0.05: Diet effect: *p* < 0.01 DF: (1,19) F: 8.65; Bonferroni post hoc: ^*^p < 0.1; 2 tailed *T*-test: ^**^p < 0.01 Aged: not significant; DF: (1,11) F: 1.03; Bonferroni post hoc: ^**^ *p* < 0.01: 2 tailed *T*-tests: ^**^p < 0.01 ^***^p < 0.001. Scale = 200 μm.

We have previously shown that a diet enriched with AXT can attenuate MPTP induced neurotoxicity [34]. Here, we corroborate our previous experiments and show that AXT supplementation can preserve the population of TH containing neurons in the substantia nigra pars compacta in young animals. As expected, we observed a significant reduction in TH positive neurons in the SNpc of MPTP injected mice that consumed the standard NIH rodent diet (CTL) compared to their saline injected counterparts. In contrast, the animals that were fed the AXT diet were protected from this loss of TH immunoreactivity (Figure 1A, 1B; F (1,19) = 8.65, *p* < 0.01). In the aged animals, we observed a similar impact of MPTP toxicity as compared to the young. However, we did not detect a protective effect of AXT on TH levels in the SNpc in the aged animals (Figure 1C, 1D, F = 1.03, DF (1,11), ns). This disparity does not seem to be due to an age related interruption in the absorption of dietary AXT (Table 1). In fact, according to HPLC analysis, both young and aged animals that received saline injections have similar levels of AXT in the plasma, suggesting that this dose of AXT did not show any differences in bioavailability or absorption in the control mice. Plasma concentrations of AXT have only a weak relationship to the TH levels in the SNpc when compared across both age groups in the MPTP treated mice (*r*^2^ = 0.07), indicating that any variability reflected in the AXT serum levels following MPTP had minimal effect on its actual neuroprotective capacity for TH in neurons.

**Table 1:** Astaxanthin concentration in the plasma (ng/mL)

Group	Average	Standard Dev.	*N*
Young NaCl	47	6.7	6
Young MPTP	61	11.3	9
Aged NaCl	40	4.1	7
Aged MPTP	41	4.5	11

**Figure 2:**
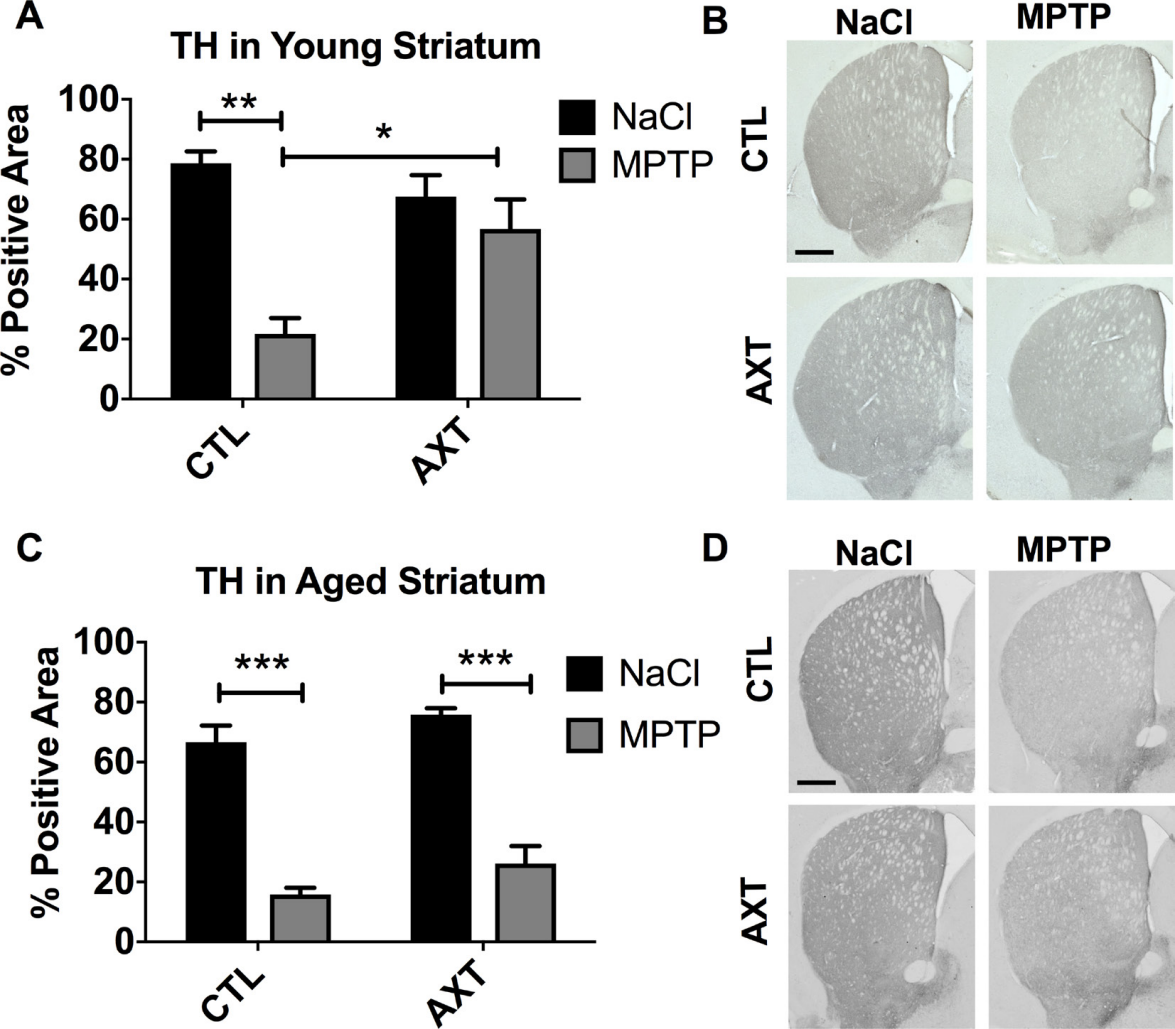
Consumption of the AXT enriched diet protects against the loss of TH positive fibers innervating the striatum in young mice However, aged mice show similar levels of denervation after MPTP regardless of the diet. A bar graph of the positive area of striatal levels of TH in the young mice (**A**) and aged mice (**C**). Data is represented as mean + SEM. Immunohistochemistry with anti-TH antibody in the striatum of (**B**) young and (**D**) aged mice. 2 way ANOVA; Young: *p* < 0.05, DF (1, 13) F: 6.4; 1 Bonferroni post hoc: ^**^p < 0.01, 2 tailed *T* test: ^*^p < 0.05; Aged: not significant; DF: (1,29) F: 0.02: Bonferroni post hoc: ^***^p < 0.001 Scale = 200 μm.

Consistent with previous reports, we show a similar effect of striatal TH preservation in young animals that were pretreated with the AXT enriched diet (Figure 2A, 2B). As expected, we measure a significant reduction in TH immunoreactivity after MPTP injections in animals that were fed the CTL diet. Animals that were fed a diet supplemented with AXT retained more TH immunoreactivity after MPTP exposure compared to controls (F (1,13) = 6.4, *p* < 0.05). However, AXT supplementation did not effectively rescue the TH immunoreactivity in the aged animals, which showed similar levels to the MPTP CTL diet group (Figure 2C, 2D; F = 0.02 DF (1,29) n.s.).

**Figure 3:**
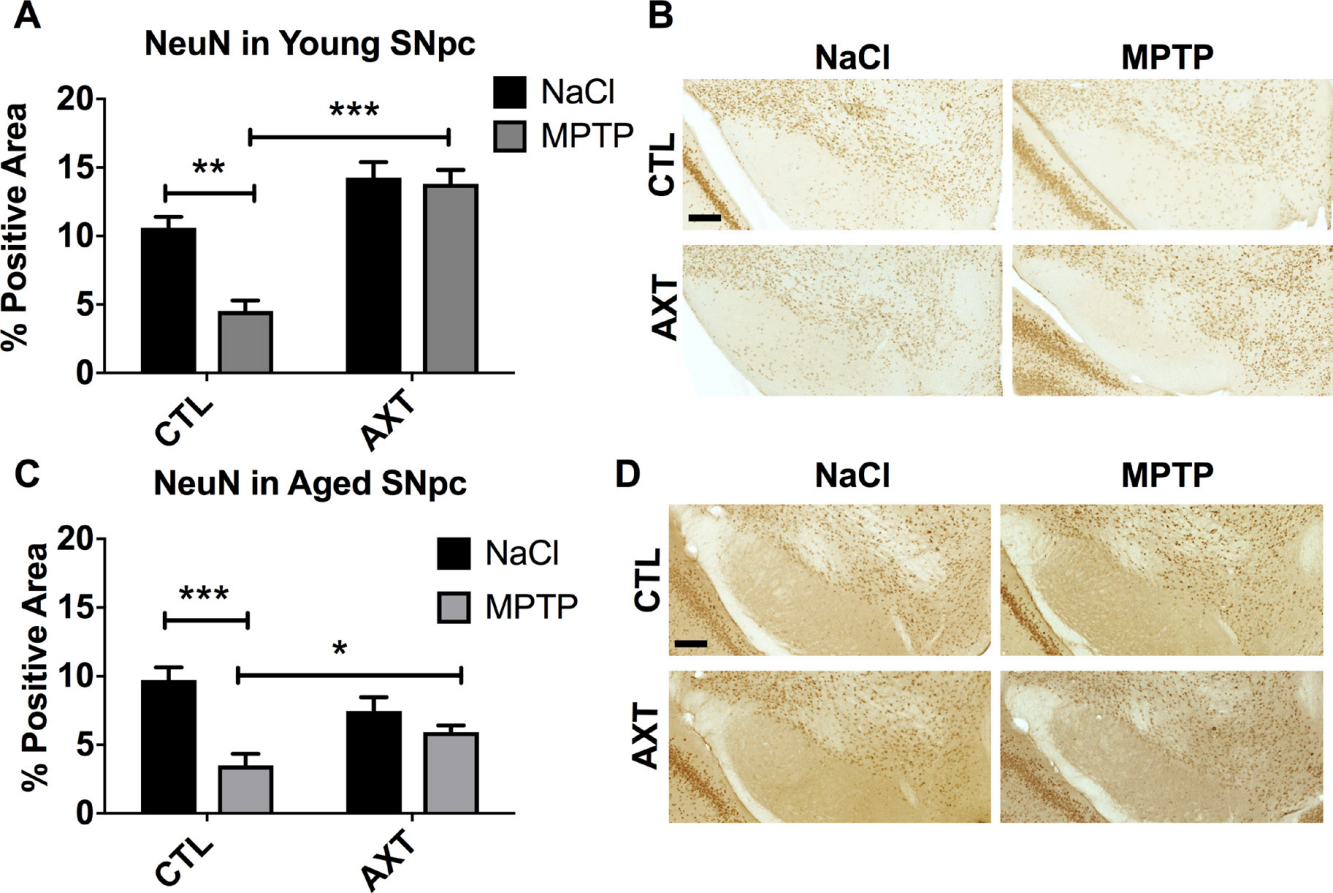
AXT enriched diet protects against neuron loss in the SNpc exposed MPTP Both young and aged mice that were treated with AXT show a retention of NeuN positive neurons in the SNpc despite the MPTP insult. A Bar graph of percent area of positive staining of NeuN relative to a user defined threshold in (**A**) young mice and (**C**) aged mice. Data is represented as mean + SEM. Representative images of immunohistochemistry with anti-NeuN antibody in the SNpc of (**B**) young and (**D**) aged mice. 2 way ANOVA; Young *p* < 0.05, DF: (1,11) F: 8.26; 2 tailed *T*-tests: ^***^p < 0.001; Aged: *p* < 0.05 DF: (1,29) F: 7.48; 2 tailed *T*-test: ^*^p < 0.05 Scale = 200 μm.

MPTP is a mitochondrial toxin and leads to the excessive release of reactive nitrogen species. Therefore, the MPTP toxin can immediately alter the expression of the tyrosine hydroxylase protein through nitrative modifications, without overtly leading to dopaminergic cell death. In this case, without neurodegeneration, it has been observed that short term reduction of TH can eventually be recovered in this experimental model. We quantified neurons by staining for neuronal nuclei (NeuN) in order to further assess the degree of neuron loss vs TH loss. We observe that the expression TH reflects the retention of NeuN in the young animals (Figure 3A, 3B). As expected, the different dietary conditions alone do not seem to alter the number of neurons in the SNpc. However, exposure to the MPTP toxin reduces neurons in the SNpc of animals that were fed the CTL diet compared to the saline injected controls. As expected from our previous work, AXT supplementation protected against this MPTP induced neurodegeneration in the young animals (Figure 3A, 3B; F (1,11) = 8.26, *p* < 0.05). Interestingly, we also observed this protective effect against neuron loss in the aged animals (Figure 3C, 3D; F (1,29) = 7.48, *p* < 0.05). We did observe an overall reduction in NeuN in the AXT supplemented aged mice that were injected with MPTP, but there was a significant rescue of NeuN staining in animals that were fed AXT compared to the control diet animals. Suggesting that there was a dietary neuroprotective effect against widespread cell death in the SNpc of the aged animals, but this effect was not sufficient to rescue levels of TH after MPTP.

### AXT alters microglial response to MPTP neurotoxicity

**Figure 4:**
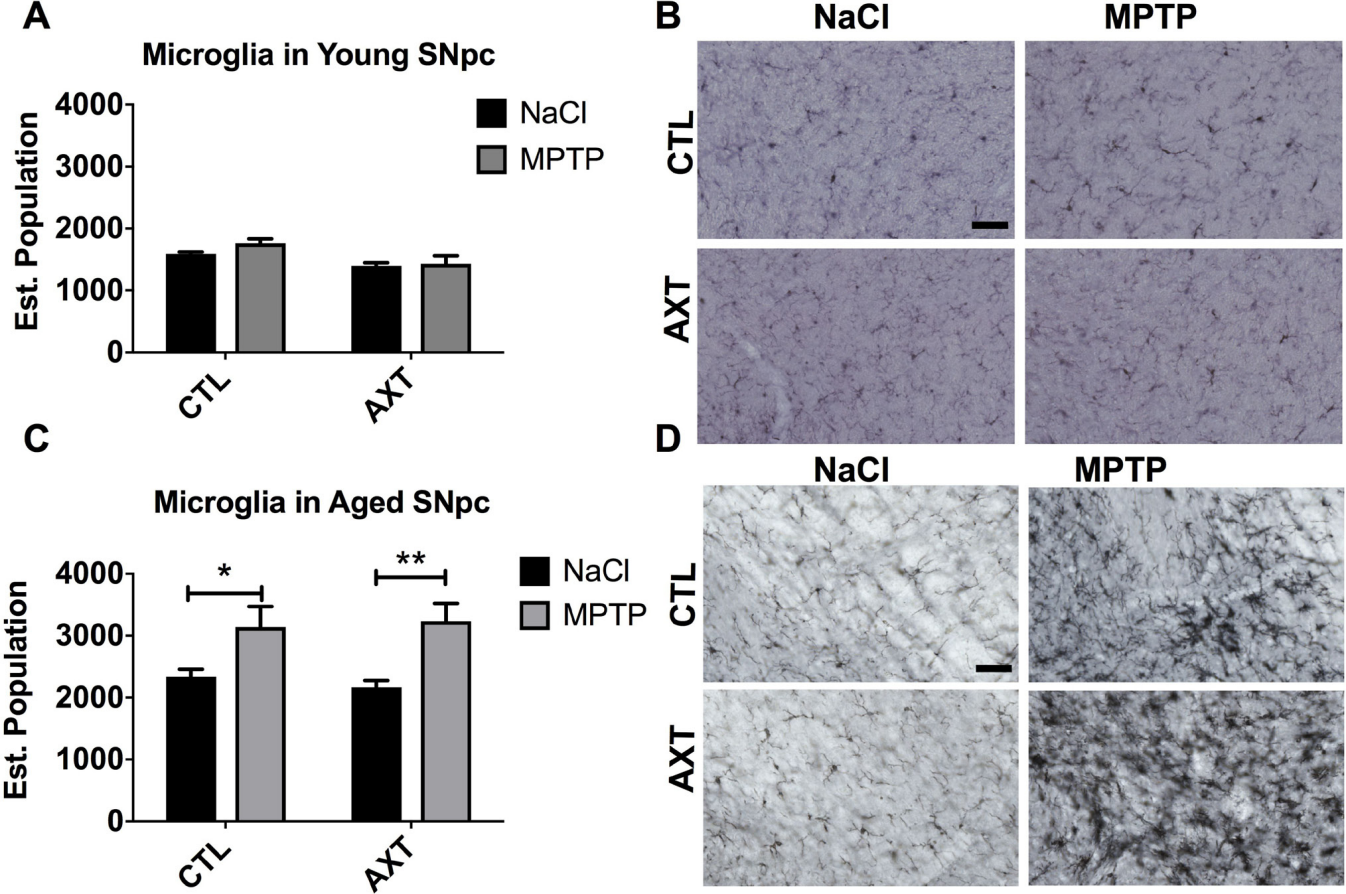
Exposure to MPTP increases the number of IBA 1 positive microglia in the SNpc of aged but not young mice (**A**) Bar graph of the estimated population of IBA 1 positive microglia in the SNpc. Data is represented as mean + SEM. 2 way ANOVA; Young: not significant: DF: (1,19) F: 0.58; Aged: not significant: DF: (1, 27) F: 0.33; 2 tailed *T*-test: ^*^p < 0.05, ^**^p < 0.01. Immunohistochemistry with anti-IBA1 antibody in the SNpc of (**B**) young and (**D**) aged mice. Scale = 50 μm.

**Figure 5:**
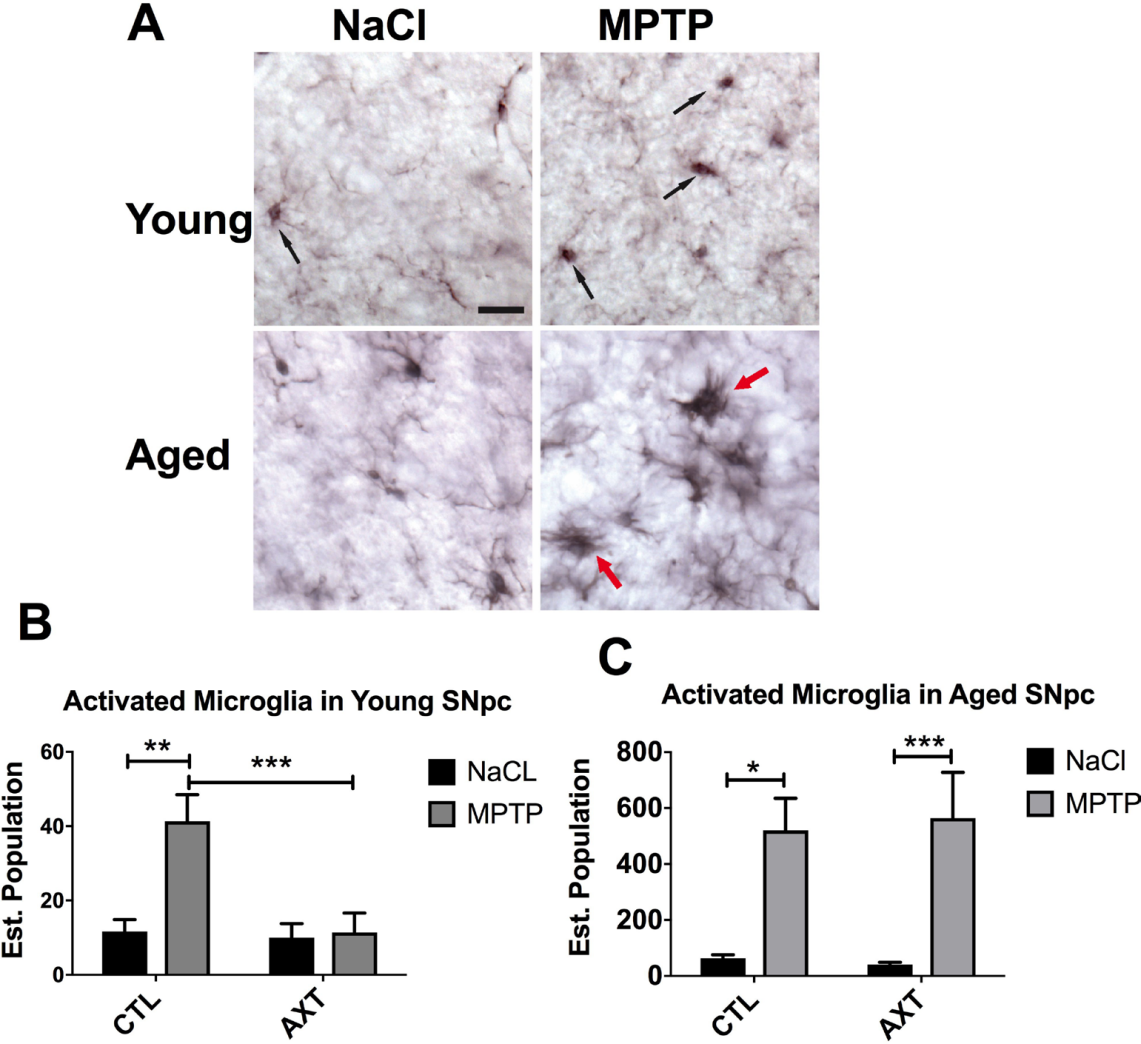
AXT supplementation decreases MPTP induced microglial activation in the SNpc of both young and aged mice (**A**) Representative immunohistochemistry depicting cells that were categorized as activated in both young and aged animals. A bar graph of estimated population of activated microglia cells in the SNpc of (**B**) young and (**C**) aged mice. Data is represented as mean + SEM. aged mice. 2 way ANOVA: Young: ^*^p < 0.05, DF: (1, 18) F: 7.03: 2 tailed *T*-test: ^***^p < 0.001; Aged: not significant, DF: (1,26) F: 0.01; 2 tailed *T*-tests: ^*^p < 0.05, ^***^p < 0.001.

Neuroinflammation is a major contributing factor in the neurodegenerative process that underlies PD. Multiple reports suggest that AXT has anti-inflammatory properties [24, 31, 41, 42]. In the brain, the inflammatory response is predominantly carried out by microglia. Therefore, we investigated the microglial response to MPTP neurotoxicity across dietary conditions. Microglia are motile and can infiltrate areas of neuronal damage to phagocytose degenerating neuronal material and initiate the repair process. We examined the number of microglia in the SNpc by counting the number of IBA 1 positive cells in the region. Stereological analysis of the young animals shows there are similar levels of IBA 1 positive cells in the nigra after MPTP exposure, suggesting that the MPTP lesion does not significantly impact microglial migration to the area of injury. We observed a trend for the dietary treatment with AXT moderately decreases the number of microglia in the SNpc after MPTP injections compared to those on the CTL diet, however, this shift is not statistically significant (Figure 4A, 4B). Based on these observations alone, it is still unclear if the AXT has this effect because it acts directly on the microglia, or because it is neuroprotective, and indirectly reduces the neurodegeneration that recruits microglia to the site of damage. However, in the aged animals we observed that the MPTP exposure increases the number of microglia in the SNpc in both dietary conditions F (1, 27) = 0.33, despite the initial hypothesis that AXT supplementation should attenuate microglial activity (Figure 4C, 4D). It is possible that the mechanisms through which AXT is modulating microglial activity may be complex. In order to help clarify this issue, we next assessed microglial phenotype and morphology using unbiased stereology to count microglial cells that matched size and intensity criteria for activation. Because age itself induces some degree of microglial priming (discussed above), microglia in the aged animals are generally larger, darker and less ramified. Furthermore, microglia of the aged animals treated with MPTP clearly show a classic hyper-reactive response to the toxin. In contrast, the microglia in the young animals are characterized by smaller cell bodies, and have maintained their branched processes. Microglia in the young animals were generally phenotypically very different from those in the aged mice; they appear to have recovered more substantially from the MPTP insult, consistent with previous reports [43]. Because of these differences, we screened for activation compared to a baseline cell size and staining intensity of microglia in each age group. In the aged condition, cells qualified as activated if they were over 8 microns large (this was easily measured using the size of the cursor in the computer software, at a 20× objective), as well as hypertrophic (Figure 5A, red arrows). In the young animals, cells were considered activated if the cell body measured 4 microns in size and black in color (Figure 5A, black arrows). This was determined by matching the soma to the inner portion of the same cursor. As expected, our stereological estimates indicate MPTP stimulates microglial activation in the SNpc. Dietary treatment of AXT reduces microglia activation in young animals (Figure 5B; F (1,18) = 7.03, *p* < 0.05). However, aged animals exposed to MPTP show an increase in the activated phenotype in both of the dietary treatments; thus, AXT did not seem to attenuate microglial activation in the aged mice (Figure 5C; 5F (1,18) = 0.01, *p* < 0.05).

**Figure 6:**
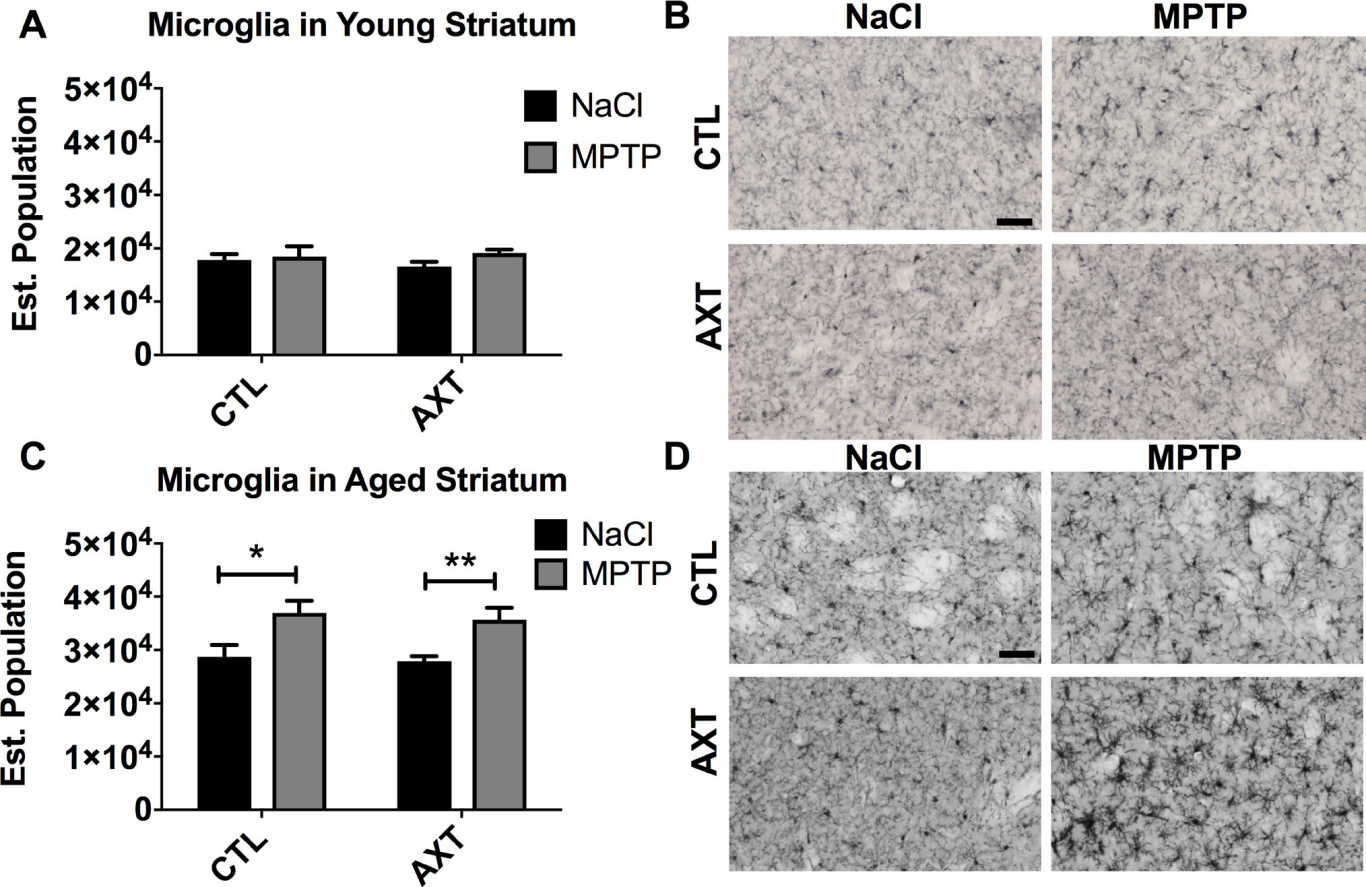
In young animals, the number of microglia in the STR was not altered by MPTP or the AXT treatment However, the aged animals show a significant increase in microglial migration to the MPTP lesioned area that was not attenuated by the AXT supplementation. A bar graph illustrating the estimated population of IBA 1 positive cells in the striatum of (**A**) young and (**C**) aged mice. (A) 2 way ANOVA not significant: DF (1,25) F:0.59 (C) 2 way ANOVA not significant, DF 1,29 F: 0.02 2 tailed *T*-test ^*^p < 0.05 ^**^p < 0.01. Data is represented as mean + SEM. Representative images of immunohistochemistry with IBA 1 in the striatum of (**B**) young and (**D**) aged mice.

We observed similar microglial responses in the striatum. In the striatum of young animals, the number of microglia did not change significantly, likely because there was less neurodegeneration in these animals compared to their aged counterparts (Figure 6A, 6B). In contrast, we detected substantially more microglial infiltration in the striatum of aged animals that were injected with MPTP and the dietary treatment of AXT did not significantly decrease the number of microglia in the striatum compared to the numbers observed in the CTL diet animals (Figure 6C, 6D; F (1,29) = 0.02, *p* < 0.05).

## DISCUSSION

PD is very prevalent neurodegenerative disease and its association with aging is clearly defined [2–6]. Current treatment strategies are inadequate and developing disease modifying agents capable of slowing or restoring neurodegeneration is an important, unmet need in the field. The xanthophyll carotenoid AXT has recently been implicated as a possible disease modifying agent in the treatment of PD [34]. It is postulated that this compound in particular is able to interrupt multiple mechanisms involved in the neurodegenerative cascade that occurs in PD, including neuroinflammation, nitrative or oxidative stress, and features mitochondrial dysfunction including impaired maintenance of the membrane potential [28, 29, 31, 42, 44–46]. The capacity for AXT to intervene at various levels that feed into neurodegeneration, for it to ameliorate multiple pathological mechanisms is a clear advantage of using AXT therapeutically. Furthermore, this naturally occurring molecule has been established as relatively safe for human consumption; there have been little to no reported adverse events attributed to AXT supplementation [47, 48]. While the extent of drug interactions has not yet been fully elucidated, AXT may be considered for long term use, possible as a preventative or adjuvant therapy.

Here we used naturally derived astaxanthin, to attenuate the neurodegeneration caused by MPTP exposure, a dopaminergic cell toxin, in both young and aged mice. Our data show that AXT is successful in reducing the neurotoxicity in the MPTP model of PD, but has limited efficacy in the aged mammalian system. As previously reported, we show that an AXT enriched diet can effectively protect against the loss of TH positive cells in the substantia nigra pars compacta of young animals that were exposed to the MPTP neurotoxin. However, when injected with MPTP, the aged mice treated with AXT showed similar reduction in TH positive neurons compared to the mice who consumed the CTL diet. Similar trends were observed in the striatum. Young mice treated with AXT were protected from the MPTP induced neurodegeneration of dopaminergic projections, while mice on the standard diet demonstrated a typical reduction in TH immunoreactivity in the striatum. In contrast, the aged animals were not substantially protected by the AXT diet; aged animals that were supplemented with AXT show only a marginal retention of TH in the striatum after exposure to MPTP compared to the mice on the standard, control diet, although this effect was not statistically significant. It has been shown that aged animals are more susceptible to MPTP, and that the neurotoxin causes a more dramatic reduction of TH in the aged brain [49]. Again, this highlights the importance of the aging process and its interaction with the underlying pathophysiology of Parkinson's disease. These observations may be partly due to this increased vulnerability in the aged microenvironment. It is possible that AXT was not able to fully protect against the loss of TH from MPTP toxicity because the same dose of MPTP is more potent in the aged animals than in the young. The use of equivalent concentrations of MPTP across age groups allows for direct comparison of lesion development and recovery, despite the heightened sensitivity of the aged animals to MPTP. However, it would be informative for future studies to experiment with a dose response to MPTP in order to elucidate a lesion size that AXT could rescue. Likewise, it would also be advantageous for future studies to explore a range of doses of AXT that may increase recovery from neurodegeneration.

It is known that MPTP can cause a temporary decrease in TH expression without overt cell death [50]. For this reason, we also quantified the neuronal nuclei to determine if the loss of TH coincided with the loss of neurons in the SNpc. In the young animals the changes in NeuN expression corroborate the trends in TH, suggesting that MPTP injections caused neurodegeneration in the mice that were fed the normal, control diet, but AXT treatment preserved the neurons in the SNpc. Interestingly, we also observed a rescue of NeuN positive cells in the aged animals fed the AXT diet compared to the control diet. This indicates that although there is still a decrease in TH after MPTP in the aged animals, AXT may still offer some neuroprotective effect in the aged mice. Often loss of TH is transient and it is possible that the aged DA neurons had not yet fully restored the TH content at 7 days following MPTP. Date *et al.* (1990) demonstrate that young mice will almost completely recover TH levels across different brain regions within three months, while aged mice do not recover in the same time frame [51]. While these researchers used a higher dose of MPTP in their experiment (4 × 20 mg/kg), it is possible that the differences in protective capacity of AXT between young and old mice was related to the duration of our study. If we allowed animals to recover from MPTP toxicity for a longer period of time, we may have observed a dietary treatment effect in TH levels as well as that observed for NeuN. It is also possible that a higher dose of AXT is needed in the aged mice, as there are differences in how the aged mice respond to MPTP as discussed above. However, it is our belief that the AXT mediated retention of neuronal nuclei is indicative of surviving DA cells in the nigra, thus leaving the potential for significant recovery of DA transmission.

We also report that AXT supplementation can influence the microglial response to MPTP induced neurotoxicity, as AXT treated mice demonstrate less microglial activation in both age groups. Data from the current study corroborates preliminary studies also indicating that AXT may modulate the inflammatory reaction to various CNS insults [24, 27, 42]. For example, Yook *et al.* (2016), observed reduced transcription of proinflammatory mediators in mice treated with AXT [52]. Other studies corroborate this shift of transcription of inflammatory cytokines, and show that AXT can also suppress TNF-a expression by blocking the translocation of NFκB to the nucleus [27]. It has also been shown that animals treated with AXT have a reduced inflammatory response to LPS administration, and are protected from the damage that occurs from the inflammatory sequelae of this robust inflammogen [53]. These observations are noteworthy because, while the pathophysiology that underlies neurodegeneration in PD is complex, modulating microglial activity has been clearly shown to be a successful and viable therapeutic strategy. For example, it is known that compounds including fractalkine [54] or the Rho Kinase inhibitor Fasudil [55] that can directly act on microglia and modulate their response have been effective in preclinical studies at mitigating the neurodegeneration in an MPTP model. This suggests to us that the trends we observed in IBA 1 staining are directly related to the neuroprotective effect of AXT in the young animals.

However, the aged animals show a different microglial response to the AXT. This may be due to a number of factors as the normal aging process includes a number of physiological changes, many of which may influence the mechanisms of action of AXT. It is unlikely that this disparity in neuroprotection is due to changes in AXT's antioxidant capacity, but it is possible that AXT is no longer efficacious at this dose, in the aged environment, because oxidative damage has been accumulating over time. Mamun Al-Amin *et al.* (2015) demonstrated that age does impact the efficacy of AXT in the brain's endogenous antioxidant systems. These authors assessed the levels and activity of antioxidant enzymes across major brain structures in young and aged animals. They also illustrated that AXT was more beneficial in fortifying the endogenous antioxidant system in young brains, while this effect was blunted in the aged microenvironment [56]. It is also likely that the lack of neuroprotection against loss of TH in the aged animals is directly related to the age-related changes of microglial activity and response to the MPTP. It has been established that microglia become primed in the aged environment, and not only demonstrate higher activation at baseline, but are also simultaneously hyper responsive to stimuli and resistant to the regulatory signals to restore microglia function to a physiological state [16]. The later may explain why we do not observe a reduction in microglial activation with AXT in the aged animals. MPTP is a very potent neurotoxin and it rapidly produces a dramatic lesion (a frequent criticism of this model), thus, it does not accurately represent the time course and degree of neurodegeneration that occurs in the human condition [57]. Aged animals are thought to be more susceptible to this toxin [58, 59], therefore, it is possible that this is not the best suited animal model of PD to fully elucidate the therapeutic potential of AXT in an aged population. Given that the aged microenvironment is more sensitive to CNS insults, it may be better to use a slower acting pathological mechanism to model PD, such as the AAV mediated over expression of synuclein. This model may be better to explicate the efficacy of AXT, or other natural compounds, in interrupting the interaction of age and the pathophysiology of PD.

In conclusion, we have corroborated our previous findings that AXT is neuroprotective against MPTP induced neurotoxicity in young animals. We show that AXT preserves TH in the striatum and substantia nigra of young animals exposed to MPTP, but the same protective effect for TH was not observed in the aged mice treated with AXT. Interestingly, we did observe a neuroprotective effect in the aged mice when we examined anti-NeuN staining, suggesting that we could preserve neuron loss but not loss of TH. We establish here that aged animals respond differently to both the MPTP toxin and the AXT supplement. This disparity in neuroprotection suggests that some of the normal changes that occur in aged animals, are impactful in the response to proposed novel therapeutic compounds. Given the parallel changes in SNpc neurons in both normal aging and in Parkinson's disease, it has been postulated that aging creates a “pre parkinsonian state”. This concept is highly impactful, because it suggests that only using young mice to model PD pathology ignores a vital contributing element that drives the disease process in the human conditions. By using aged animals, we capture a more realistic physiological environment that includes an underlying vulnerability. Ultimately, investigating these pathological mechanisms in an aged mammalian system should increase translatability of preclinical findings into useful medications or treatment strategies. Future studies are needed to further assess the disparity in AXT neuroprotection in young and aged animals. It is possible that AXT was less efficacious in aged animals simply because of the dose used. It is also possible that aged animals require a higher dose in order to prevent the robust microglial activation and oxidative stress that occurs after MPTP. Additionally, there may be other physiological changes brought about during the normal aging process that effect how AXT functions. Given that we observed a treatment effect of AXT in preserving neurons but not TH, it is likely that the time points in our experiment did not include the full recovery from the MPTP insult in the aged animals. It is possible that we may see an improvement in TH levels with AXT treatment if the recovery time was extended.

## METHODS

All procedures were conducted according to the National Institute of Health Guide and Use of Laboratory Animals and the University of South Florida IACUC. Male C57BL/6J were used in these studies at both 3 and 18 months of age. Animals were housed in groups of 4 and maintained in controlled conditions of 21°C and a 12 hr light/dark cycle. The rodent diets were administered daily by an experimenter in order to ensure freshness of the food and added test compounds, while the animals fed *ad libitum.* In order to confirm that the mice were consuming the enriched diets, research staff regularly recorded the food intake and body weight of the mice and did not observe any significant differences in weight gain over the course of the experiment due to the dietary treatments.

### Dietary pretreatment

Mice were treated with Bioastin^®^ generously supplied by Cyanotech to achieve a dose of 30 mg/kg bodyweight, based on average daily consumption of a standard rodent diet. Bioastin^®^ is a proprietary product designed to deliver algal derived, natural astaxanthin powder derived from *Haematococcus pluvialis* on a microbead. These microbeads are comprised of inert ingredients gelatin, corn starch, sucrose and facilitate the dispersion of astaxanthin in water. These microbeads comprise a high volume of the total Bioastin^®^ product, therefore we included a separate vehicle control diet in previous experiments to demonstrate that the microbeads alone did not have a discernable bioactivity. We have previously shown that the vehicle control diet does not have an effect. These compounds were incorporated into the Harlan Teklad rodent diet, and delivered *ad libitum.* Mice were fed the AXT enriched diet for 1 month before MPTP administration, and dietary treatment was continued throughout the duration of the experiment, for 5 weeks total of AXT supplementation. Food intake per cage and individual body weights were monitored throughout the course of treatment to ensure consumption.

### MPTP administration

The MPTP HCL (Sigma-Aldrich) was diluted in sterile saline and injected intraperitoneally. The mice received four injections at a dose of 10 mg/kg, administered once per hour for four hours for a final dose of 40 mg/kg MPTP. Because aged mice may be more susceptible to MPTP toxicity, this dose was pilot tested in a separate aged cohort to ensure that it would not cause excessive mortality. Mice in the control condition received equal volumes of sterile saline delivered in the same time course. Mice were allowed to recover from MPTP exposure for 7 days before they were fully anesthetized with phenobarbital and transcardially perfused with PBS. Brains were immediately removed and one hemisphere was microdissected for the structures of interest. The other hemisphere was preserved in 4% paraformaldehyde for 24 hours. The brains were then transferred to a 30% sucrose solution for cryoprotection.

### Immunohistochemistry

Paraformaldehyde fixed brains were treated with a 30% sucrose solution for three days to ensure cryoprotection before 40 μm thick slices were prepared using a cryostat. Every 6th section was selected for consistent sampling for IHC procedures, including extra sections outside of the regions of interest to ensure thorough sampling and analysis. Free floating sections were blocked in goat serum before being incubated in primary antibody (TH: Immunostar 1:1,000, IBA1: Wako Laboratory Chemicals 1:2,500, NeuN: Millipore 1:5,000). Primary antibodies were diluted in PBS containing goat serum and Triton X-100 overnight at 4°C while the sections were incubated in secondary antibody for 60 minutes at 25°C. Avidin-biotin complex (Vector Labs) was used to increase substrate formation from the precipitation reactions were developed with diaminobenzidine (Sigma-Aldrich).

### Quantification

Estimated population of TH or IBA 1 positive cells was determined using the optical fractionator probe and unbiased stereological methods from Microbrightfield (Cholchester VT). Substantia nigra was sampled with a counting frame of 150 × 150 and grid size of 200 × 200 μm, while the striatum sampled with the same size counting frame and a grid size of 300 × 300 μm. The area of positive staining of each antibody of interest (TH or NeuN) was quantified using a digital image analysis program, NearCYTE (nearcyte.org), previously described [60]. Briefly, high resolution images were created with the AxioScan microscope at a 20× objective. Using NearCYTE software, regions of interest were traced over the digital slide images, then compared to a standard of intensity dictated by an experimenter blind to treatment conditions. This comparison essentially generates a ratio of the number of pixels that match this user defined criteria within the region compared to those that fall below the threshold. It is necessary to evaluate the immunoreactivity of TH in the striatum in this manner, as this region consists of cross sections of dopaminergic terminals yielding a diffuse staining pattern. However, we have previously determined that data collected with NearCYTE and expressed as a percent positive area also reliably and accurately corroborates the data collected with unbiased stereology and presented as an estimated population of positive cells within a region [60].

### High-performance liquid chromatography

In order to verify that the experimental conditions (age or injections) did not differentially affect AXT absorption, we evaluated the levels of AXT in the plasma by HPLC using a liquid-liquid extraction method. Briefly, 100 μL of plasma was spiked with 100 μL acetone containing 1 μg/mL of testosterone as an internal standard and vortexed for 5 minutes at room temperature. 200 μL of N-hexane was added, vortexed again for 5 min at room temperature, then centrifuged at 14000 rpm at 25°C. The upper organic layer was transferred to a 96 well plate and dried under gentle nitrogen stream. The dried extract was reconstituted with 100 μL of MeOH.

100 mL of the resulting extract was injected on a Thermo HPLC system equipped with PAL CTC plate sampler (96-well plate), Dionex Ultimate 3000 binary pump (flow rate at 1 mL/min), Dionex Ultimate 3000 thermostatted column compartment (temperature at 40°C), Thermo Endura Mass Spectrometer (ESI source), using Phenomenex Develosil^®^ (3 μm, 4.6 × 50 mm, 140Å) column under isocratic condition of MeOH aq (0.1% formic acid) starting at 98% during 0.5 min, then under gradient condition of 98–100% MeOH aq (0.1% formic acid) over 4 min, finishing at 100% of MeOHaq (0.05% formic acid) over 0.5 min then returning to 98% MeOH aq for another 1 min to re-equilibrate. For quantification, trans-astaxanthin was diluted in DMSO then spiked into plasma to generate calibration curve.

Plasma levels of AXT were determined by calculating the area of the peak relative to the AXT calibration curve. AXT level was adjusted to the internal standard concentration to correct for analyte lost during the extraction process. Peak height measurements were conducted referring to values obtained for standards of known concentrations.

### Data analysis

The data presented graphically as the group mean and standard error of the mean. Statistical analysis was performed using GraphPad Prism software. 2 Way ANOVAs were conducted, while Bonferroni multiple comparisons posthoc tests (unless otherwise specified) were used to further compare differences between groups.

## Abbreviations

PD: Parkinson's disease
AXT: astaxanthin
MPTP: 1-methyl-4-phenyl-1,2,3,6-tetrahydropyridine
SNpc: substantia nigra pars compacta
STR: striatum
TH: tyrosine hydroxylase
NeuN: Neuronal Nuclei
IBA1: Ionized calcium binding adaptor molecule 1
DA: dopaminergic
